# Smallpox, Monkeypox and Other Human Orthopoxvirus Infections

**DOI:** 10.3390/v15010103

**Published:** 2022-12-29

**Authors:** Galina A. Shchelkunova, Sergei N. Shchelkunov

**Affiliations:** State Research Center of Virology and Biotechnology “Vector”, Rospotrebnadzor, Koltsovo, 630559 Novosibirsk, Russia

**Keywords:** variola virus, monkeypox virus, orthopoxviruses, evolution, diagnostics, vaccine, chemotherapy

## Abstract

Considering that vaccination against smallpox with live vaccinia virus led to serious adverse effects in some cases, the WHO, after declaration of the global eradication of smallpox in 1980, strongly recommended to discontinue the vaccination in all countries. This led to the loss of immunity against not only smallpox but also other zoonotic orthopoxvirus infections in humans over the past years. An increasing number of human infections with zoonotic orthopoxviruses and, first of all, monkeypox, force us to reconsider a possible re-emergence of smallpox or a similar disease as a result of natural evolution of these viruses. The review contains a brief analysis of the results of studies on genomic organization and evolution of human pathogenic orthopoxviruses, development of modern methods for diagnosis, vaccination, and chemotherapy of smallpox, monkeypox, and other zoonotic human orthopoxvirus infections.

## 1. Introduction

Based on the report of the Global Commission for the Certification of Smallpox Eradication of the World Health Organization (WHO) (December, 1979), it was solemnly proclaimed at the 33rd World Health Assembly (WHA) on 8 May 1980 that all the nations of the Earth have defeated this especially dangerous infection. In order to prevent accidental spread of the causative agent of this infection from laboratories storing samples of variola (smallpox) virus (VARV), measures have been applied to reduce the number of these laboratories. In 1975 there were 75 laboratories storing VARV in different countries. By 1984, however, there remained only two laboratories that received the status of WHO Collaborating Centers for Smallpox and Other Poxvirus Infections—the Institute for Viral Preparations (IVP), Moscow, USSR and the Centers for Disease Control and Prevention (CDC), Atlanta, Georgia, USA [1,2].

Although tightly controlled by WHO, these stocks of viable VARV strains were considered a potential biohazard. Therefore, at the fourth meeting of the Ad Hoc Committee on Orthopoxvirus Infections in Geneva in March 1986, the following solution was proposed: “The Committee reviewed the need for retaining the remaining stocks of variola virus. It was noted that the variola gene pool could be cloned into non-expressing sites in bacterial plasmids, for future studies of variola virus. … Because implementation of a decision to destroy all variola virus stocks could be irrevocable, some 60 virologists working in 21 countries had been consulted by a Committee member before the meeting; only 5 thought that variola virus should be maintained indefinitely. Taking these facts into consideration, the Committee concluded that the cloned DNA provided sufficient reference material to resolve any future diagnostic problem involving suspected smallpox and that research studies of variola requiring culture of the virus were no longer justified. Thus, in the Committee’s opinion, there was no need to retain stocks of viable variola virus any longer.” [3].

In December 1990, the 5th meeting of the Ad Hoc Committee on Orthopoxvirus Infections reported the following: “All stocks of variola virus and materials containing variola virus must be destroyed by December 1993.” Given the planned destruction of collections of VARV strains, it was important to reliably conserve the genetic material of different VARV isolates in a biologically safe form. In order to preserve information about this unique virus, the Committee considered it necessary to carry out preliminary studies on genome sequencing. National VARV genome sequencing programs proposed by the Russian Federation (IVP, Moscow and the State Research Center of Virology and Biotechnology (SRC VB) VECTOR, Koltsovo, Novosibirsk region) and the United States (Center for Disease Control and Prevention (CDC), Atlanta, Georgia and the Institute for Genomic Research, Gaithersburg, Maryland) were approved; and a Technical Committee was established to oversee these DNA sequencing efforts [4].

By the middle of 1992, Russian researchers were the first ones to complete the genome sequencing and the bioinformatic analysis of a highly virulent VARV major strain isolated in India in 1967 during an outbreak of smallpox with a case-fatality rate of 31% [5,6,7]. The study results were first reported at the 9th International Conference on Poxviruses and Iridoviruses (Les Diablerets, Switzerland) in September 1992 [8]. Later, American researchers completed whole-genome sequencing of another highly virulent VARV major Bangladesh-1975 strain, isolated during a smallpox outbreak with a mortality rate of 18.5% [9,10].

After publication of the report of the WHO Committee on planned destruction of VARV stocks, there has been a debate about the necessity of these measures among the scientific community. In particular, during the IXth International Congress of Virology held in Glasgow in August 1993, a special plenary session was held; however, no consensus was reached on this issue [11,12].

At the 6th meeting of the Ad Hoc Committee on Orthopoxvirus Infections (9 September 1994, Geneva, Switzerland), it was decided to postpone the destruction of VARV stocks for some time and store the collections of DNA and plasmid-cloned fragments of VARV genome in two international repositories: SRC VB VECTOR (Russia) and CDC (USA) [13]. In addition, it was recommended to additionally perform sequencing and analysis of the whole genome of low-virulent VARV minor Garcia-1966 strain, which was subsequently conducted in a joint study by Russian and American research groups [14].

Taking into account the potential hazard of working with viable VARV in Moscow, VARV stocks were transferred from IVP (Moscow) to the SRC VB VECTOR (Koltsovo, Novosibirsk region) on September 27, 1994 based on a joint order of a number of ministries of the Russian Federation and the Russian Academy of Medical Sciences. Following the inspection of the highest biosafety level laboratory planed for VARV research, by the WHO panel in 1995, the WHO officially established the WHO Collaborating Center for Orthopoxvirus Diagnosis and Repository for Variola Virus Strains and DNA at the SRC VB VECTOR in 1997. The right to store VARV stocks and their genomic DNA at the SRC VB VECTOR was approved by the WHA resolution 49.10 and then confirmed by WHA resolutions 52.10, 55.15, and 60.1.

On 25 May 1996, the 49th WHA adopted a recommendation to postpone the date for possible eradication of VARV stocks to 30 June 1999 [15]. As a wide range of researchers got involved in discussion about the need to eradicate VARV strains stored at two official WHO-designated repositories, it became increasingly clear that a final decision on eradication of this unique virus should not be rushed.

In 1999, the WHO Advisory Committee on Variola Virus Research was established. The Committee began to supervise VARV studies and hold annual meetings of the scientists involved in the study of this virus and development of the methods for diagnosis, prevention, and treatment of smallpox and other human orthopoxvirus infections.

Proponents of the concept of compulsory eradication of the existing two official VARV repositories proceeded from the fact that destruction of these stocks would solve the issue of potential danger of virus release into the environment. The drawback of this viewpoint is that there is no confidence in unintentional or intentional informal conservation of VARV strains in any country. For example, forgotten glass ampoules with live VARV, which had been stored for more than 60 years, were found in a low-temperature refrigerator at the National Institutes of Health (USA) in 2014 [16]. Phenomenal advances in synthetic biology, including recent genomic DNA synthesis and revitalization of horsepox virus [17], show that VARV can also be synthesized *de novo*. All this makes the proposed destruction of VARV stocks pointless and indicates the need to intensify research to develop new methods for diagnosing, preventing, and treating not only smallpox but also other human orthopoxvirus infections.

## 2. Human Orthopoxvirus Infections

The genus *Orthopoxvirus* of the Poxviridae family includes human pathogenic species *Variola virus* (VARV) and related zoonotic *Monkeypox virus* (MPXV), *Cowpox virus* (CPXV), *Camelpox virus* (CMLV), and *Vaccinia virus* (VACV) [1,2].

Zoonotic orthopoxviruses are usually isolated from animals that are in close proximity to humans (cows, buffaloes, camels, monkeys, etc.) and serve as an intermediate host of the virus, whose natural reservoir is often other wild animals. Therefore, the name of orthopoxvirus species usually does not indicate the real animal that is the natural virus reservoir.

### 2.1. Variola (Smallpox) Virus

VARV, the causative agent of smallpox, can only infect humans; there is no natural reservoir (other susceptible animal) for this virus. Until its eradication, smallpox was one of the most dangerous human infectious diseases. At least 400 million people died from this infection in less than 80 years in the 20th century, when mass vaccination and intensive anti-epidemic measures against smallpox were carried out [18]. Two subtypes of smallpox are distinguished based on clinical manifestations: variola major, a common disease with a mortality rate of 5–40%; and variola minor, a mild disease form with 0.1–2% mortality [1].

Geographical variations with different mortality rates were found among variola major epidemics. The Indian subcontinent and adjacent areas had the highest mortality rate (up to 40%) in the second half of the 20th century, while significantly lower mortality rates (5–15%) were observed in Indonesia and Western, Central, and Eastern Africa [2]. The reasons for such a difference still remain unclear. Epidemics of variola minor in the 20th century were characteristic of North and South America, as well as South Africa [1].

The severity of smallpox infection depends on the age of the infected person. Analysis of the outbreaks of variola major in the 20th century showed that the mortality can exceed 40–50% in unvaccinated children < 5 years of age and adults > 40 years of age, while a lethal outcome is much less common (5–15%) in unvaccinated people aged 10–30 years [19].

Smallpox is a highly contagious infection: it is only slightly inferior to measles and chicken pox in this parameter. The pharynx/nasopharynx and the respiratory tract are the entry gate for infection. VARV can also enter the body through damaged skin. The secondary attack rate of variola major infection in close contact averaged > 58% for susceptible individuals. The data of the smallpox eradication program demonstrated that one patient infected an average of five contacts; although the real number was often much higher (up to 20–35 people) [1,2].

### 2.2. Monkeypox Virus

MPXV causes a human infection that clinically resembles discrete smallpox and sometimes (up to 10% of cases) causes death [1,2]. Unlike smallpox, monkeypox (mpox [20]) is usually characterized by lymphadenopathy in addition to the typical rash. The first case of mpox in humans was registered in 1970 by the staff of IVP (Moscow) during analysis of the clinical sample from an infected child in Zaire (now the Democratic Republic of the Congo, DRC) [21,22,23]. The intensive research conducted under the auspices of the WHO in 1981–1986 showed that human mpox is a rare sporadic disease resulting from animal-to-human transmission of MPXV in the rainforest areas of Central and West Africa. The natural reservoir of MPXV are various African animal species, mainly rodents. In addition, lethal mpox cases in humans were detected only in Central Africa [24]. Further genomic analysis of MPXV isolates from different African regions revealed two subtypes of MPXV: MPXV clade I (Central African) and MPXV clade II (West African) [25,26]. Experimental infection of susceptible animals showed that the MPXV clade I variant is more pathogenic than MPXV clade II [27,28,29].

An important difference between human mpox and smallpox was considered to be the low efficiency of human-to-human transmission of MPXV, which until recently prevented the transition of local outbreaks of the disease into widespread epidemics. However, recent studies have indicated an increasing efficiency of MPXV spread in the human population. According to the WHO, the number of people infected due to human-to-human transmission was 29.6% of the total number of registered cases by the end of the first Monkeypox Project (1986). The ratio changed dramatically by 1997 (73.0%). The number of identified generations in the chain of human-to-human transmission of mpox has also increased to eight. Analysis of the available data made it possible to conclude that mpox incidence in humans in the DRC (Central Africa) had increased 20 times by 2010 (30 years after cessation of smallpox vaccination) [30].

An expansion of the distribution of human mpox has been recently noted in Africa. According to the WHO, confirmed cases of mpox in humans and animals have been found in the Central African Republic, Cameroon, DRC, Liberia, Nigeria, the Republic of the Congo, and Sierra Leone in 2010–2018 [31].

The first mpox outbreak in humans outside the African continent was registered in 2003. The causative agent of this infection was introduced into the USA by infected animals imported from Ghana for sale as pets [32]. The MPXV clade IIa subtype has been confirmed to have caused the outbreak. This may explain the absence of deaths among 72 individuals infected with MPXV during this outbreak in the USA.

A human mpox outbreak in Nigeria has recently attracted attention. A total of 414 suspected cases of mpox in humans, including 152 laboratory-confirmed cases, were identified in 2017–2019. The largest number of patients were aged 21–40 years. Mpox deaths were reported in 7 cases, with 4 patients being co-infected with HIV [33,34]. Viral DNA sequencing confirmed that MPXV clade IIb caused the outbreak [26]. These are the first reports of lethal infections in humans for MPXV clade II.

The important characteristics of this mpox outbreak in Nigeria were the following: the infection has repeatedly spread outside Africa, and mpox has been reported in travelers from Nigeria to Israel in September 2018, to the United Kingdom in September 2018, December 2019, and May 2021, to Singapore in May 2019, and to the USA in July and November 2021. Moreover, one of these patients infected a healthcare worker with mpox in the UK [35,36].

After the low virulent MPXV clade IIb spread in the gay, bisexual, and other men who have sex with men (GBMSM) community, a new stage of virus evolution began. After the mass gay pride parade in Gran Canaria on 5–15 May 2022, human mpox cases were registered in many countries on different continents, with the vast majority of them among the GBMSM population [37]. The sudden appearance of human mpox in several nonendemic countries indicates that there may be a period of undetected transmission, as well as recent expansion events [38]. By 31 October 2022, more than 76,000 confirmed cases of human mpox have been registered in 102 countries where this disease was not previously detected. The widest distribution of human mpox is noted in the USA (>28,000 cases) [39].

Such a widespread mpox epidemic in humans may result in accelerated evolution of MPXV and emergence of virus variants with greater efficiency of human-to-human transmission and/or increased pathogenicity [40]. Therefore, much attention is now being paid to the control of this epidemic [41].

### 2.3. Cowpox Virus

Cowpox in humans is a sporadic disease that occurs when CPXV is transmitted from an infected animal (usually pets) to humans. CPXV is relatively low pathogenic to humans; however, it has a very wide range of susceptible animals. CPXV infection could be fatal in immunocompromised individuals [42,43] and for the foetus [44]. The disease is predominantly reported in Europe [45,46,47,48,49]. CPXV has also been found in rodents and humans in different geographic regions of Russia [2,50].

### 2.4. Vaccinia Virus

Recently, the incidence of orthopoxvirus infection caused by zoonotic VACV (buffalopox virus belongs to the same species) has also significantly increased in farm animals (cows, horses, and buffaloes) and humans in India, Brazil and Colombia [51,52,53,54,55,56,57,58,59,60].

Determination of VACV origin is one of the most complicated issues. This virus has been used for many decades to vaccinate people against smallpox; this “variolae vaccinae” virus was consider to derive from CPXV introduced into the practice of smallpox vaccination by Edward Jenner in 1796 [1]. It was only in the 20th century that it became clear that orthopoxvirus strains used for smallpox vaccination differ significantly in their properties from natural CPXVs isolated from cows and from other orthopoxviruses studied by that time [61]. Therefore, they were assigned to a separate species of *Vaccinia virus* [1,2]. At the same time, the natural reservoir of VACV remained unknown, and numerous hypotheses were proposed about the emergence of this virus during the passages of precursor viruses on animal skin when preparing smallpox vaccine samples [1,62].

The question of VACV origin was somewhat clarified after the complete *Horsepox virus* (HSPV) genome had been sequenced. Phylogenetic analysis of the conserved region indicated that HSPV is closely related to sequenced isolates of VACV and rabbitpox virus, clearly grouping together these VACV-like viruses [63]. After that, attention was drawn to the fact that E. Jenner mentioned that he often used the infectious material obtained from horses and inoculated in cows for his smallpox vaccine [64,65]. In this regard, one can assume that VACV originates from zoonotic HSPV, which persisted in nature in parallel with CPXV [40]. Apparently, not CPXV but HSPV isolates, the descendants of which were assigned to a separate species of *Vaccinia virus* decades later, were widely used for smallpox vaccination in the 19th century [66]. A recent phylogenetic analysis of complete genomes of orthopoxvirus isolates obtained in Europe from animals and humans showed that two completely distinct species, namely CPXV-like and VACV-like, coexist in nature on this continent [64].

### 2.5. Camelpox Virus

In terms of its biological properties and phylogenetic analysis of the complete viral genome sequence, CMLV is closest to VARV compared to other orthopoxviruses [67]. It had been generally accepted that the CMLV host range was limited to one animal species: camels. However, the first camelpox cases in humans have been laboratory confirmed in India and Sudan [68,69].

## 3. Evolution of Orthopoxviruses

The most direct and effective method to study the evolution of closely related viruses is whole genome sequencing and computer analysis of the obtained data. In order to establish evolutionary relationships between different human pathogenic orthopoxviruses after having sequenced the genomes of the first viruses CPXV [70] and MPXV [71,72] isolated from infected people, we performed a comparative analysis of complete genomes of VARV, MPXV, CPXV, and VACV. The analysis revealed that CPXV DNA is not only the longest genome among the studied orthopoxviruses, it also contains all the genetic elements characteristic of other species of orthopoxviruses. VARV, MPXV, and VACV can be considered CPXV variants with species-specific deletions, rearrangements, and point mutations in the genome. Therefore, we concluded that the CPXV-like virus was the progenitor of all modern orthopoxviruses pathogenic to humans [70,73].

The accumulated data made it possible for the first time to conduct a comparative analysis of the genomes of all species of human pathogenic orthopoxviruses, perform the first phylogenetic study of this group of viruses, and reveal the evolutionary relationships between them. We have found for the first time that West African and South American VARV strains form a separate subtype (clade) with significant differences in their genome organization from all other studied geographic VARV variants [74]. At the same time, a fundamental discovery was that West African and South American VARV strains form two clearly distinct phylogenetic subgroups (subclades) within the identified clade, which indicates their independent evolution. Based on the analysis results and archival data on repeated introductions of smallpox from West Africa to South America in the 16th–18th centuries during transportation of slaves, we made the first quantitative assessment of the rate of poxvirus evolution [75]. Subsequent sequencing of complete genomes of a large set of VARV strains isolated in different geographic regions in different years made it possible to clarify the periods of key events in VARV evolution [76,77].

Based on the analysis of available archival data on smallpox epidemics, history of ancient civilizations, and the latest data on evolutionary relationships between orthopoxviruses, we hypothesised that smallpox could have emerged repeatedly in the past as a result of evolutionary changes in the zoonotic progenitor virus and disappeared due to the insufficient population of isolated ancient civilizations [78]. The only historically long-lasting pandemic was the last smallpox pandemic, which was eradicated in the 20th century by combined efforts of physicians and scientists from many countries under the auspices of the WHO. There are no fundamental barriers for re-emergence of smallpox or a similar human disease in the future as a result of natural evolution of currently existing zoonotic orthopoxviruses.

## 4. Species-Specific Orthopoxvirus DNA Diagnostics

Despite the fact that orthopoxvirus infections have characteristic visual manifestations (skin lesions), experience shows that clinical diagnosis is often erroneous [2].

The development of the polymerase chain reaction (PCR) method has led to creation of modern approaches for detection and identification of trace amounts of microorganisms in analyzed samples with high specificity and in a short period of time. At the same time, it eliminates the need to carry out manipulations with highly dangerous live infectious agents, including VARV and MPXV.

Numerous methods have been proposed for PCR detection of different orthopoxvirus species [79,80,81,82,83,84,85,86,87,88,89,90,91,92,93,94]. However, in the case of human pathogenic orthopoxviruses, test systems that provide the possibility of genus-specific DNA identification of the analyzed virus with its simultaneous species-specific differentiation are of greatest interest [95,96,97,98]. SRC VB VECTOR employees were the first researchers to develop these test systems based on conventional multiplex PCR [95] and real-time multiplex PCR [97]. These conventional and real-time test systems were registered for medical use in Russia in October 2011 and February 2016, respectively. These test systems allow for simultaneous identification of VARV, MPXV, CPXV, and VACV in one reaction sample.

Oligonucleotide microarrays can also detect trace amounts of a test material in a sample. One of the important advantages of oligonucleotide microarrays over other methods of PCR-based diagnosis is the possibility to analyze multiple genetic loci simultaneously, which significantly increases method reliability. Various diagnostic oligonucleotide microarrays have been developed for reliable species-specific detection of orthopoxviruses [99,100,101,102]. However, these procedures are performed in specialized laboratories only and are not yet widely used.

Rapid development of sequencing technologies currently makes it possible to quickly obtain information on complete nucleotide sequence of the genome of an organism of interest. Thus, data on detected cases of unusual orthopoxvirus infections are increasingly supplemented by genome-wide sequences of these viral isolates [103,104]. The results of these studies indicate the need for further improvement of laboratory diagnostics of orthopoxvirus infections and epidemiological surveillance.

## 5. Vaccination

The most reliable way to prevent any viral disease is vaccination. Vaccination against smallpox (Latin *variola*) was the first historic example of such protection against an infectious disease.

Smallpox survivors were easily identified by the characteristic scars on their face skin (the so-called “pitted face”), which were left on the sites of pustules after the loss of dry crusts; these people became resistant to smallpox whenever a new outbreak of the disease occurred. Apparently, these observations formed the basis for inoculation of infectious material obtained by collecting skin crusts from smallpox patients into skin incisions (usually in the forearm) or intranasally made on healthy people in India and China. This procedure, called *variolation* (stands for *variola inoculation*), caused a moderately severe disease and provided further reliable protection against smallpox. However, 0.5–2% of variolated patients would die, which prevented the widespread use of this procedure [1].

In 1798, English physician E. Jenner described a new, safer procedure for protecting against smallpox [1,65]. Rural residents who got infected by animals that had the smallpox-like disease (cows and horses) were known to have pustular skin lesions on their hands; they suffered a mild infection that left scars phenotypically resembling those after variolation. In addition, people who had contracted cowpox were known to have become resistant to smallpox. In 1796, E. Jenner performed the first experiment in which an eight-year-old child was inoculated intradermally with material from a pustule collected from a cowpox-infected woman. To prove that the child had become resistant to smallpox after the infection, E. Jenner variolated the child after 6 weeks and found that the boy was resistant to this procedure.

Given these findings, to emphasize the protective effect of the used infectious entity against smallpox, E. Jenner introduced the term “*variolae vaccinae*” (Latin for *cowpox*; from Latin *vacca* (cow)) instead of the term cowpox and called the procedure “*vaccine inoculation*.” In 1803, Richard Dunning proposed the shortened term “*vaccination*.” In 1881, at the 7th International Congress of Medicine in London, Louis Pasteur suggested using the term *vaccination* for all protective immunization procedures against any infectious disease [65].

It should be noted that the kingdom of viruses was discovered only a century after introduction of smallpox vaccination into practice. Orthopoxviruses are antigenically and immunologically similar to each others, they participate in serological cross-reactions and provide immune protection. This provided a reliable way to protect against smallpox by inoculating a person with either CPXV or HSPV, which were later widely replaced by VACV resulting in less pronounced adverse reactions during vaccination. This vaccine turned out to be so effective that it allowed for smallpox eradication during mass vaccination of people and strict epidemiological surveillance under the auspices of the WHO. This is the first and so far the only example of eradication of a particularly dangerous human viral infection in the history of mankind [1,2].

The cessation of vaccination against smallpox after 1980 meant that a huge part of the world’s population is now not anymore immune to not only smallpox but also other zoonotic orthopoxvirus infections. This creates a new risk of possible circulation of zoonotic orthopoxviruses in the human population, which may lead to a change in the ecology and range of sensitive hosts for different orthopoxviruses.

Human monkeypox is of particular concern. In conditions of a long absence of vaccination of the population and a much more frequent infection in people, MPXV can acquire not only high transmissibility but also high pathogenicity, which is characteristic of VARV, as a result of natural evolution. If this happens, humanity will face a problem much more difficult than smallpox eradication. This is primarily due to the fact that, unlike for VARV, the natural reservoir of MPXV is various African rodents.

In order to prevent the development of outbreaks into widespread epidemics and thereby reduce the risk of emergence of highly pathogenic orthopoxvirus due to natural evolution, the researchers’ efforts should be focused on creation of new generations VACV-based safe live vaccines [40,78]. VACV-based vaccines do not have a pronounced species specificity against human pathogenic orthopoxviruses. Therefore, they can be used in outbreaks caused by any orthopoxvirus for immunization of both humans and animals.

The first-generation live smallpox vaccine was a VACV preparation obtained by virus propagation on the skin of calves and other animals. Its use for mass vaccination is currently limited due to the relatively large number of possible severe complications [1], which is due to the increased number of immunodeficient people in recent years.

Currently, VACV vaccine strains are produced in mammalian cell cultures, and these preparations are classified as second-generation smallpox vaccines [105]. The ACAM2000 vaccine, which was licensed for use in the USA in August 2007, is the most studied smallpox vaccine of this type [106,107]. Although cell culture-based vaccines are produced according to modern standards, both first- and second-generation smallpox vaccines can cause serious adverse reactions during vaccination and therefore have limited use.

Attenuated third-generation smallpox vaccines are created by multiple passages of a specific VACV strain in a heterologous host cell culture. For example, the most studied third-generation smallpox vaccine strain MVA was obtained after a large number of passages of the Ankara VACV strain in chicken embryo fibroblasts. The MVA strain genome contains multiple mutations and extended deletions compared to the DNA of the original VACV strain. MVA is incapable of replicating in most mammalian cells, including human cells [108].

To date, a third-generation smallpox vaccine based on the MVA-BN strain, manufactured by Bavarian Nordic (Denmark), has passed numerous clinical trials, including studies in patients with atopic dermatitis and HIV [109,110,111,112]. This two-dose live, non-replicating vaccine was approved in the EU countries in July 2013 under the name of IMVANEX, IMVAMUNE in Canada, and JYNNEOS in the USA (September 2019). The vaccine was intended for primary vaccination of patients with contraindications to first- and second-generation smallpox vaccines. This vaccine was originally approved for smallpox immunization and later expanded to be used against monkeypox as well.

The third-generation smallpox vaccine LC16m8, which was licensed in Japan, was obtained based on the VACV strain Lister by multiple passages in primary rabbit kidney cells at low temperatures (30 °C). Clinical studies of VACV LC18m8 have shown a significant reduction in side effects compared to the conventional Lister strain vaccine. The attenuation of the vaccine strain is mainly due to a mutation in the B5R gene, which encodes an extracellular virion protein. The protective efficacy of LC16m8 was shown to be comparable to that of the parental strain Lister in experiments in various animal models [113,114,115,116].

A new approach to producing fourth-generation attenuated smallpox vaccines involves the use of genetic engineering techniques to introduce targeted deletions/insertions to disrupt genes regulating the body’s defense responses against viral infection. The first variant of such VACV studied in detail is the NYVAC strain; the NYVAC genome lacks a block of 12 genes and contains 6 impaired genes. NYVAC was shown to induce significantly lower immunity against smallpox in humans compared to the conventional vaccine [117].

A highly attenuated VACdelta6 variant was obtained by sequential introduction of targeted deletions/insertions in six individual genes of the LIVP strain at the SRC VB VECTOR [118,119,120]. VACdelta6 underwent full cycles of preclinical studies and clinical trials as a vaccine against smallpox and other orthopoxvirus infections. A live VACdelta6-based culture vaccine called OrthopoxVac was licensed in Russia in November 2022. The vaccine is recommended for immunization against smallpox, mpox, and other orthopoxvirus infections.

## 6. Chemotherapy

Chemotherapeutic drugs, the search for which has gained some success in the last twenty years, may be important for the treatment of human orthopoxvirus infections. The initial screening for orthopoxvirus replication inhibitors is usually carried out in cell cultures. Since there is no adequate animal model of smallpox, testing of potential drugs against smallpox has to be performed in surrogate animal models of smallpox infection. Substances that have shown high antiviral activity in vitro are usually studied in animal models such as intranasal or aerosol infection of mice with ectromelia virus (ECTV) and CPXV and monkeys infected with MPXV [121]. Rabbits infected with the rabbit poxvirus (belongs to the VACV species) [122,123,124], as well as prairie dogs and ground squirrels (*Marmota bobak*) infected with MPXV, have also been actively used in recent years [125,126,127]. However, none of the surrogate animal models of orthopoxvirus infection fully mimics smallpox infection in humans. Therefore, putative smallpox chemotherapy drugs are being studied in different animal models in parallel.

Compound ST-246 presents the greatest interest as a therapeutic drug against smallpox, mpox, and other orthopoxvirus infections; it inhibits the last stage of enveloped virion assembly and prevents orthopoxvirus virion release from the infected cell [128,129,130,131]. ST-246 was identified during screening of a drug library consisting of >350,000 unique chemical compounds for antiviral activity in the cell culture. ST-246 showed low toxicity and high antiviral efficacy in mice infected with ECTV, VACV, and CPXV, rabbits with rabbitpox virus, prairie dogs with MPXV virus, and non-human primates with MPXV and VARV [126,129,130]. Tecovirimat SIGA (ST-246) is a medicine against smallpox, mpox, and cowpox. It is also used to treat complications arising after smallpox vaccination. In July 2018, the U.S. Food and Drug Administration (FDA) approved TPOXX (Tecovirimat), the first drug indicated for the treatment of smallpox. In December 2021, oral Tecovirimat was approved in Canada to treat smallpox. In January 2022, Tecovirimat was approved in the European Union to treat orthopoxvirus diseases (smallpox, mpox, cowpox, and vaccinia complications) in adults and in children who weigh ≥ 13 kg.

An ST-246 analogue called NIOCH-14 also showed high activity against orthopoxvirus infections in various animal models [132,133,134,135]. After the completion of preclinical and clinical trials, NIOCH-14 was approved for clinical use in Russia in October 2022.

A viral DNA polymerase inhibitor, the nucleotide analogue Cidofovir approved for clinical use in cytomegalovirus retinitis, proved to be an effective anti-orthopoxvirus drug [121]. Cidofovir had been shown to be a relatively effective therapeutic drug against orthopoxvirus infections in different animal models. However, its significant drawback was poor water solubility and the need for intravenous administration. Therefore, a lipid conjugate of Cidofovir named CMX001 (Brincidofovir) was synthesised. This broad-spectrum antiviral drug can be used in tablet form and still has a pronounced anti-orthopoxvirus activity [123,136,137,138]. In April 2021, the FDA approved Brincidofovir under the commercial name of Tembexa in tablet and oral suspension formulations to treat smallpox in all age groups, including infants, and for patients who have difficulty swallowing. If necessary, this drug can be used against human mpox.

Considering that the widespread use of antiviral drugs results in emergence of resistant virus strains, effective treatment of possible cases of smallpox, mpox, and similar orthopoxvirus infections requires further search for new chemotherapeutic drugs with different molecular targets in orthopoxviruses.

## 7. Conclusions

The cessation of smallpox vaccination more than 40 years ago and the subsequent loss of herd immunity in humans against not only smallpox but also other orthopoxvirus infections promotes an increased spread of zoonotic orthopoxviruses among people. This, in turn, can contribute to the natural selection of highly pathogenic and epidemic virus strains. The human mpox epidemic, which spread in many countries in 2022, is the first big challenge for researchers and physicians.

Considering that this might happen in the future, WHO efforts have been focused on development and testing of modern methods for orthopoxvirus identification, creation of safe new-generation smallpox vaccines, and chemotherapy drugs against VARV and other orthopoxviruses pathogenic to humans at the two WHO Collaborating Centers for Smallpox (Russia, USA) since the beginning of the 21st century [125,139]. These efforts resulted in successful developments that were licensed in several countries and provide hope for effective control and counteraction of human orthopoxvirus infections.

Nevertheless, it is important to continue research in these fields in order to create new safe and highly effective means to prevent and treat orthopoxvirus infections. Diagnostic methods should focus on rapid identification of not only VARV and MPXV but also CPXV, VACV, and CMLV. Given the increased number of outbreaks of orthopoxvirus infections in animals and humans in recent years and their potential danger, constant monitoring of these infections should be embraced in all parts of the world. This will reduce the risk of small outbreaks developing into widespread epidemics and, thereby, decrease the chance of re-emergence of a highly contagious and pathogenic orthopoxvirus. It is also important that stocks of new vaccines and chemotherapeutics should be widely available and in sufficient quantities to treat the emerging biological threats effectively.
